# A Novel Nutritional Predictor Links Microbial Fastidiousness with Lowered Ubiquity, Growth Rate, and Cooperativeness

**DOI:** 10.1371/journal.pcbi.1003726

**Published:** 2014-07-17

**Authors:** Raphy Zarecki, Matthew A. Oberhardt, Leah Reshef, Uri Gophna, Eytan Ruppin

**Affiliations:** 1 School of Computer Sciences & Sackler School of Medicine, Tel Aviv University, Tel Aviv, Israel; 2 Department of Molecular Microbiology and Biotechnology, Faculty of Life Sciences, Tel Aviv University, Tel Aviv, Israel; The Pennsylvania State University, United States of America

## Abstract

Understanding microbial nutritional requirements is a key challenge in microbiology. Here we leverage the recent availability of thousands of automatically generated genome-scale metabolic models to develop a predictor of microbial minimal medium requirements, which we apply to thousands of species to study the relationship between their nutritional requirements and their ecological and genomic traits. We first show that nutritional requirements are more similar among species that co-habit many ecological niches. We then reveal three fundamental characteristics of microbial fastidiousness (i.e., complex and specific nutritional requirements): (1) more fastidious microorganisms tend to be more ecologically limited; (2) fastidiousness is positively associated with smaller genomes and smaller metabolic networks; and (3) more fastidious species grow more slowly and have less ability to cooperate with other species than more metabolically versatile organisms. These associations reflect the adaptation of fastidious microorganisms to unique niches with few cohabitating species. They also explain how non-fastidious species inhabit many ecological niches with high abundance rates. Taken together, these results advance our understanding microbial nutrition on a large scale, by presenting new nutrition-related associations that govern the distribution of microorganisms in nature.

## Author Summary

Understanding microbial nutrition is critical for understanding microbial life, and thus has a major influence in many areas of biology. In recent years, the traditional methods of studying microbial nutrition, which rely on culturing bacteria and assessing their nutritional needs through extensive experiments, have been augmented by the development of genome-scale metabolic models, which enable in-depth analysis and prediction of nutrition for a few well-studied organisms. Recently, a pipeline was developed for generating genome-scale metabolic models automatically (the SEED). Here, we leverage models built from this pipeline in order to develop a novel predictor of microbial minimal medium requirements, which we then apply broadly for thousands of microbes across the tree of life. We first show that nutritional requirements are more similar among microorganisms that co-habit many ecological niches. We then use our medium predictions to examine the fastidiousness of organisms (i.e., their need for complex/specific media), and suggest an explanation for certain observed features of microbial abundance patterns. This study is one of the first to leverage genome-scale models on a large (>1000 species) scale, and sets the potential for a new host of strategies for understanding microbial nutrition and ecology in the future.

## Introduction

Microbial nutrition influences global ecosystems, food production, and human disease. However, most microbes in nature are difficult or impossible to cultivate, and have thus traditionally been nearly impossible to study [1]. Recent cultivation-independent methods such as metagenomic sequencing have changed this, revealing inner mechanics of microbes in samples from whole environments, even if these microbes cannot be individually accessed. This has inspired several thousand studies over the last few years, which have begun to extract order out of previously inscrutable ecosystems [2], [3], [4], [5].

Meanwhile and in parallel, broad insights into microbial lifestyles have begun to emerge through a number of new computational strategies [6], [7], [8], [9]. Particularly notable is Genome-Scale Metabolic Modeling (GSMM), which allows analysis of the full metabolic requirements of microorganisms, but until recently was restricted to a selected group of intensively studied microorganisms [10], [11]. GSMMs have been used in the past to determine potential nutrient needs of organisms, including attempts to determine minimal nutrient requirements of *Haemophilus influenza* [12] and the neglected tropical parasite *Leishmania major* [13], and critical (i.e., impossible-to-replace) functions in *Escherichia coli* [14]. Media prediction methods have also been used recently in context of automated model building efforts [15].

A breakthrough in GSMM modeling occurred with the development of model-SEED, a resource of automatically built, functioning GSMMs for thousands of microbes, ranging from the barely to the exhaustively studied (the SEED: [16], [17]). SEED models have been used to analyze metabolic capacities of dozens of species of the antibiotic-producing phylum Actinobacteria [18], [19], and have also been combined with large-scale ecological data to reveal cooperation and competition patterns among microbes, including a previously unobserved phenomenon in which unidirectional loops are formed among sets of organisms that each require a metabolite produced by another [7]. The detection of such patterns requires large scale data and the ability to integrate it in a meaningful fashion – a capability that is only now, through these new tools, becoming available.

It is in the context of these new capabilities that we use an expanded set of several thousand SEED models to investigate systems features of the ecological lifestyles of microorganisms, searching for new potential associations that relate central metabolic and ecological variables to the minimal nutritional needs of microbes. We focus on minimal needs because many microorganisms in nature are oligotrophs, i.e., they cannot survive in rich media [20], [21]. We first develop a method to predict minimal growth media as a kind of specific nutritional signature for these organisms. This method is akin to the ‘minimal reaction sets’ method of Burgard et al. [22], which determines the smallest number of metabolic reactions that can be active while still enabling biomass production over a certain target level. We apply a similar methodology but focus on minimizing the number of exchange reactions, which allows us to determine the smallest number of compounds that can be taken up by a cell while still enabling some target production of biomass. We next compare these nutritional signatures across organisms from different ecological niches, and find a striking similarity between patterns of environmental co-growth and minimal nutrient sets. We then explore our minimal nutritional predictions in the context of various genomic and environmental factors, and develop new insights into the relationships between microbial nutritional fastidiousness (i.e., the complexity and specificity of nutritional needs of an organism), environmental versatility, interspecies cooperation, and environmental co-growth. These insights hint at fundamental patterns that might govern microbial growth, and lay a foundation for future development of model-guided chemically defined growth media.

## Results

### *MENTO*, a predictor of microbial nutritional fastidiousness

In order to relate microbial nutrition to ecological distributions and lifestyles, it was first necessary to develop a nutritional predictor that could be universally applied across many organisms. Keeping in mind that many (perhaps most) microbes in nature are oligotrophs [23], we chose to focus on the *minimal* nutritional needs of microorganisms. Developing a minimal medium predictor in itself is an important basic question that could have a broad and significant impact in microbiology [21].

To develop such a predictor, we leveraged genome-scale metabolic models from SEED and developed a new algorithm, the Minimal ENvironment TOol (MENTO), to predict minimal nutritional requirements for microorganisms. MENTO employs a mixed-integer linear programming (MILP) algorithm with a GSMM in order to determine the least number of compounds possible to be in an environment while still enabling growth of a specific microorganism. Production of a nominal level of biomass is enforced during this procedure, which ensures that all of the components needed for cell growth can be produced (see methods). The number of individual compounds in a minimal medium computed by MENTO is a natural measure of nutritional fastidiousness, as a higher number of compounds in the computed environment/medium denotes that an organism is more fastidious, i.e., that it requires a larger set of specific nutrients to survive (and hence is a nutritional specialist, whereas less-fastidious organisms, which would typically have small MINENVs, are nutritional generalists).

We observed that minimal environments computed by MENTO contain two types of metabolites: a unique core of *critical metabolites* that must be present in *any* predicted minimal or non-minimal growth media environment for a given microorganism, and then a periphery of ‘replaceable’ metabolites that provide essential elements to the microorganism in the given solution but may not appear in other, equally minimal (in terms of total number of nutrients) media solutions. To standardize predicted minimal environments for cross-species analysis, we therefore developed a set of *unique* minimal environment predictions for each species (hereafter referred to as MINENVs), which contain the number of compounds predicted by MENTO to be the minimal possible, but preferentially include low molecular weight compounds for nutrient sources (this is achieved by solving a second MILP problem, which reduces the total molecular weight of compounds in an organism's MINENV, while not exceeding the number of compounds predicted by the first MILP optimization in MENTO – see Methods).

The MINENVs were thus predicted aspiring to find the *simplest* basic compounds that organisms can be predicted to grow on. All subsequent analyses are based on these unique, simplified MINENVs unless otherwise noted. Overall, MENTO was applied to compute the constitution of the MINENVs and critical metabolites for 2529 microorganisms in SEED, and the results can be seen in Tables S1–S3, with an analysis of the essentiality of the different components provided in Figure S1 in File S3. We found these MINENVs to be relatively invariant to small changes in a metabolic model (single gene removals in *E. coli* only altered the minenv in 6% of cases, and changed the size of the MINENV in only 4% of cases), and so serve as a reasonable yardstick for comparison across species.

### Comparing predicted MINENVs to known experimental lab media

Media have been developed in the past for culturing a large number of microorganisms in the lab. To test and validate our minimal environment predictions, we reconstructed known compositions of defined media for a subset of microorganisms in our dataset from published media in the Leibniz Institute DSMZ media/strain collection (see Methods). We then compare our predicted minimal environments to these known experimental lab media to determine if MINENVs we predict follow the trends of actual medium compositions.

**Figure 1:**
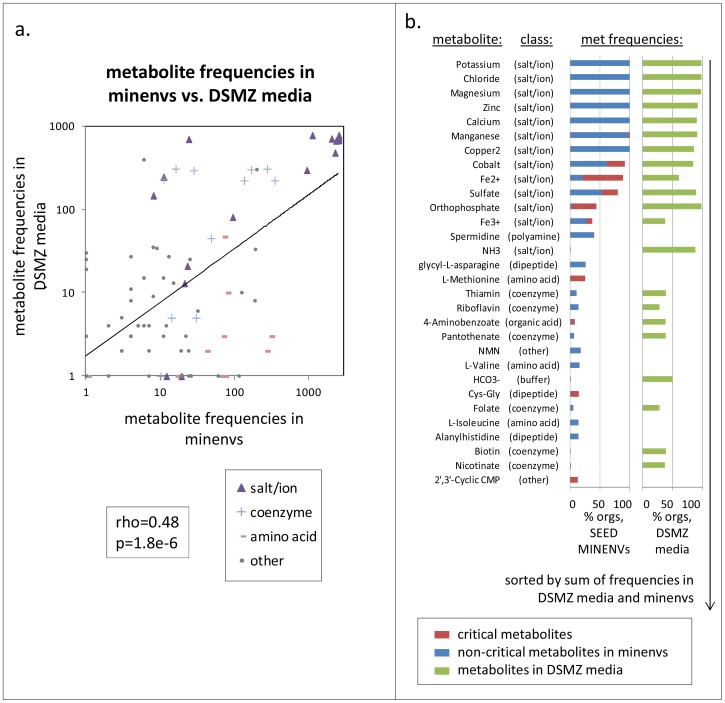
Metabolite prevalence in u-minenvs across organisms. (A) Frequencies of appearance are compared for all metabolites shared between the DSMZ known lab media and minenvs. Each metabolite is classed into one of 4 categories, as noted, and a trendline is shown. (B) The most common metabolites in minenvs and DSMZ media are listed, along with their compound class and their frequency in both spaces. Metabolites are sorted based on the sum of DSMZ media usage and minenv usage.

It was important to first understand the degree to which compounds we predicted to occur in MINENVs are common in established lab media, and to understand where possible discrepancies lay. We therefore compared the frequency of appearance of metabolites in the MINENVs we had computed versus the frequency of their appearance across all fully defined media in the DSMZ media database, a set that includes 791 distinct media. Metabolites shared between MINENVs and the DSMZ media were more abundant in both datasets than metabolites that were not shared (p = 5.6e-5 and p = 7.6e-8 in ranksum tests on frequencies in MINENVs and DSMZ media, respectively). Furthermore, among the 90 metabolites shared between the two datasets (out of 227 in DSMZ and 372 in the MINENVs), the frequency of metabolite appearance across the two spaces correlates significantly (Spearman rho = 0.48, p = 1.8e-6; see Figure 1a).

To better understand the roles of different metabolites, we manually classified metabolites in the set shared by the DSMZ media and the MINENVs into typical biological and chemical roles (non-defined media components, such as ‘yeast extract’ were excluded from this analysis). Notably, the most prevalent metabolites in both datasets are metal ions, followed by coenzymes (see Figures 1a and 1b). Nucleic acid compounds, such as nucleotides and nucleosides, tend to appear frequently in MINENVs but not in DSMZ media, as do amino acids (Figure 1a), whereas simple alcohols tend to be more prevalent in DSMZ media than in MINENVs. Amino acids also came up as the most differentiating metabolites between organisms living in different ecological environments and lifestyle categories (see analysis in File S1). These observations point to areas in which SEED metabolic models should be refined, but also areas of potential improvement in developing oligotrophic lab media, such as the increased usage of amino acids. The full results of this analysis are provided in Table S1 in File S2.

There was of course no guarantee that the MINENVs we predict, which are not unique among minimal media, would look like the lab media, which are chemically defined but are not necessarily minimal. Nevertheless, we expected that there would be some trend of similarity of MINENVs and DSMZ media per microorganism. To directly test this, we recalculated MINENVs for each microorganism, this time preferentially choosing compounds that are indeed present in the DSMZ media collection as nutritional sources for that strain (see methods). Comparing the re-calculated MINENVs versus the DSMZ media for all 71 DSMZ lab media on which organisms in our MINENV dataset grow, we found that MINENVs are closer to the proper DSMZ medium per microorganism than expected by chance (p = 0.003 in a non parametric test of # significant p-values across the set of 71; see methods). This small but significant trend indicates that the calculated MINENVs do capture some of the DSMZ features. However, more work will be required in the exchange capabilities of the SEED models before they are able to fully and reliably recapitulate known lab media.

### MINENVs are predictive of viable media compositions, but are not highly selective between organisms

To assess the predictive potential of the MENTO algorithm, we used MINENV predictions to form minimal lab media for each of 5 commonly grown heterotrophic bacteria: *Escherichia coli*, *Agrobacterium tumefaciens*, *Bacillus subtilis*, *Pseudomonas aeruginosa*, *and Serratia marcescens*. We transferred the predictions to lab-realizable media using them as a base, and considering typical metabolite concentrations from M9 minimal medium (e.g., see formulation of P. aeruginosa medium in Figure S4a–b in File S3). We then experimentally grew each of these microorganisms in microwell batch cultures in each of the media to assess the computational predictions (See Figure S4c in File S3 and methods). Reassuringly, each microorganism grew on the medium based on its own MINENV prediction. However, we far under-predicted growth of microorganisms on media not designed for them (only 6 of our 17 negative predictions turned out to be correct). This result may partially be a reflection of an intrinsic bias towards nutritional flexibility among heterotrophic microorganisms (such as the bacteria that we tested), but it also may point to trend of higher true nutritional flexibility than our models predict.

To check whether inaccuracy in the predictions may have arisen because of errors in the SEED models, we also assessed growth *in silico* in human curated models of *E. coli*, *B. subtilis*, and *P. aeruginosa* on the five lab media. These models predicted significant growth under many more media conditions than the SEED models (consistent with what was seen *in vitro*), but displayed less precision, and were not overall better predictors (see Figure S4c in File S3). However, the higher numbers of positive growth phenotypes among these models supports the hypothesis that the SEED models tend to be more pessimistic predictors of growth, and thus that they may require more improvements before they can reliably predict the selectivity of new media. It is also acknowledged that even in the best studied bacteria, not all nutrient scavenging pathways have been characterized [24], and the automatic gap-filling of SEED models is focused on biomass production but does not attempt to incorporate all potential carbon sources, as this would often require addition of an unacceptably large number of reactions to fill gaps [16].

### Metabolic requirements are more similar among environmentally co-growing organisms

**Figure 2:**
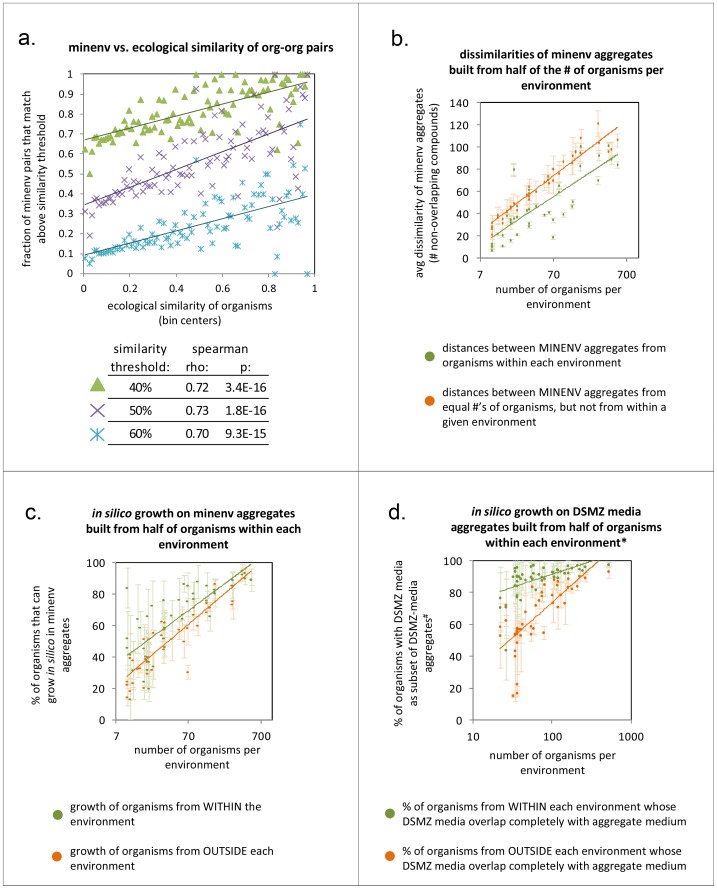
Nutritional requirements are more similar among ecologically co-distributed organisms. (A) Similarity of ecological distributions (in Greengenes) and also similarity of MINENVs were calculated for each of ∼3 million organism-organism pairs, using jaccard metrics (see methods). Org-org pairs were then binned by ecological distance (x-axis), and the ratio of pairs in each bin with a MINENV similarity above some percentage threshold (i.e., 100*jaccard similarity) was determined (y-axis). A range of MINENV similarity thresholds were explored; three are shown on the plot, along with the correlation coefficients between ecological and MINENV similarities. Each dot on the plot represents all org-org pairs falling into a given ecological distance bin. (B) microorganisms from within each Greengenes environment are split into two groups 100 times, and distances between aggregate minenvs for each group are compared (each green dot is an environment; see methods). This was repeated each time for the same number of organisms but not from within the given environment (orange dots). Errorbars denote standard deviations over multiple trials and dots denote means. (C) FBA is performed to determine what percentage of organisms from within an environment (green dots) or outside an environment (orange dots) are able to grow *in silico* on aggregated MINENVs built from half of the organisms within an environment (organisms used to build environments are never used for the test). (D) The test of growth from (C) is repeated, but aggregate environments are composed from unions of DSMZ media of 50% of organisms from within an environment, and ‘growth’ is assessed by whether an organism's DSMZ medium is contained fully within the environmental aggregate.

Organisms that co-grow in an ecological environment must make do with the same set of nutrients. Therefore, we expected that organisms sharing many ecological niches would have similar minimal nutritional needs. To test this, we mapped microorganisms from the SEED database to operational taxonomic units (OTUs) from Greengenes, a database of ecological distributions of microbes. Distributions of microorganisms as reported in Greengenes have been grouped previously into ecological environments (see [7], [25], and methods). We binned organism pairs based on similarity of their ecological distributions, and within each ecological distance bin, we assessed the percent of organism pairs whose MINENVs are similar above some threshold (MINENV and greengenes similarities were calculated by Jaccard metrics; see methods). Using these criteria, we found a strong correlation between similarity of MINENVs and similarity of ecological distributions for organism-organism pairs [e.g., ρ≥0.70, p<1e-14 in Spearman test with MINENV similarity thresholds of 30–70%; see Figure 2, and methods; if we removed insufficiently sampled ecological distance bins (i.e., those with less than 1000 org-org pairs out of the possible ∼3 million), we obtained significant correlations even up to MINENV similarity threshold of 99% (rho = 0.54, p = 1.9e-4)].

Having established this trend, we next explored the association between nutritional needs of organisms and co-growth within individual environments. To do this, we formed “aggregated” MINENVs for groups of microorganisms by taking the union of their MINENV metabolites, i.e., by including in an aggregate MINENV all compounds present in all of the MINENVs of organisms making up the aggregate. We observed that aggregate MINENVs computed for two groups of microorganisms from within the same environment tend to be more similar than aggregate MINENVs computed from two random sets of organisms (p = 3.8e-3 in t-test for lower average distance of MINENVs of within- vs. outside-environment organisms across 43 environments, using half the number of organisms per environment to form groups; see Figure 2b and methods; also re-confirmed using only 5 organisms to form the aggregate MINENVs, as shown in Figure S5 in File S3). Finally, we observed that organisms from within an environment grow *in silico* on the aggregate media of that environment more often than randomly picked organisms (see Figure 2c; p = 3.9e-2 in t-test for difference in survival using FBA, and p = 8.3e-12 for a model-free test using DSMZ media, as shown in Figure 2d and explained in methods). Taken together, these results indicate that the minimal nutritional needs of organisms correlate strongly with ecological co-growth, and highlight this property as a potential avenue for developing new growth media for yet uncultivable microorganisms.

### Metabolic requirements reflect the breadth of ecological distributions

**Figure 3:**
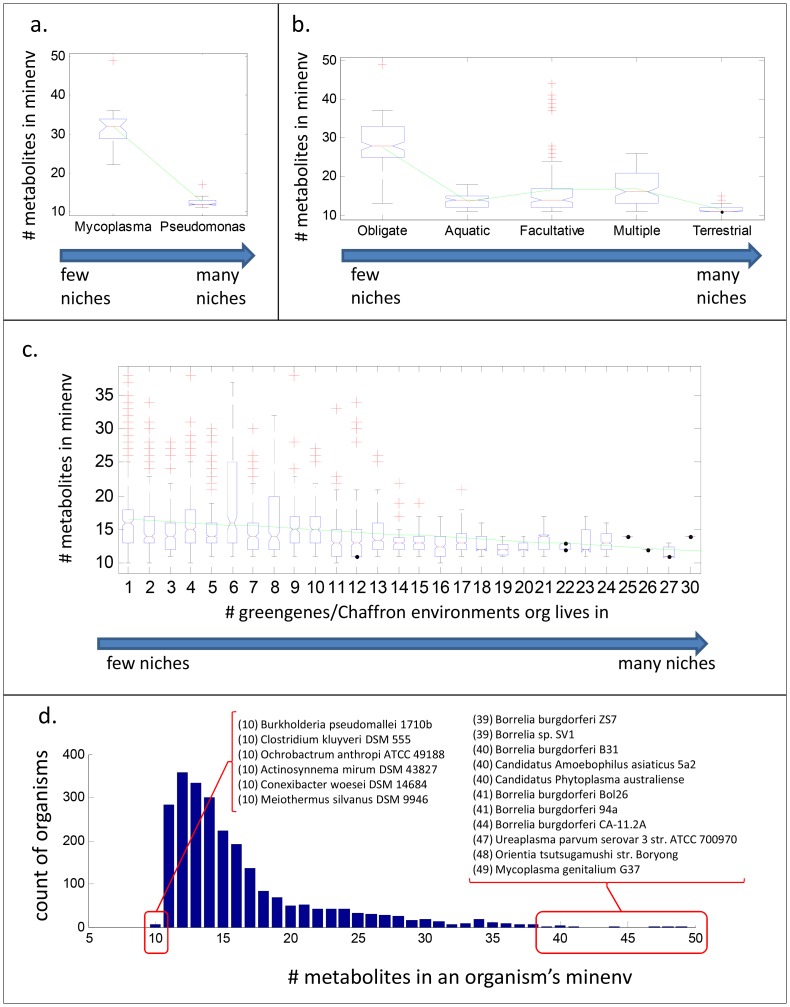
Nutritional fastidiousness scales negatively with breadth of ecological environments. This trend is shown (A) between species of *Mycoplasma* and *Pseudomonas*, (B) between five lifestyle categorizations with ascending environmental breadth, and (C) in environmental distributions in Greengenes. (D) shows a histogram of the number of organisms with different sized minenvs. Species on the extreme ends are listed.

The size of a given MINENV denotes the minimal number of distinct metabolites an organism needs in order to proliferate. Thus, MINENV size of a given organism can be interpreted as a measure of its nutritional fastidiousness, which is a useful yardstick for comparison with other features of microbial lifestyle. One trend we were interested in investigating is the relationship between fastidious growth requirements and environmental versatility, with the hypothesis that more fastidious microorganisms will display less diversity in the environments they live in than less fastidious ones. Certain strong exemplars of this hypothesis exist, including the obligate parasitic species *Mycoplasma*, which is known to require extensive nutrition to survive, and the notoriously ubiquitous species *Pseudomonas aeruginosa*, which can survive on a minimal salts medium using a wide variety of single carbon sources [26], [27]. As a starting point, we compared the number of compounds present in the MINENVs of 25 *Mycoplasma* species versus 28 *Pseudomonas* species in our data set, and observed reassuringly that *Pseudomonas* species have many fewer MINENV components than *Mycoplasma* species (medians = 12 and 32 metabolites, respectively; ranksum test p = 3e-10; see Figure 3a).

To check if our predicted MINENVs show this trend across a broader range of microorganisms, we next mapped microorganisms in our study to a previously developed set of 5 lifestyle categories, whose rank reflects the breadth of environments each microorganism can live in, with higher rank indicating more breadth (categories are based on and updated from [28]; see Table S6 in File S2). Against this categorization, we obtained a significant negative correlation vs. the number of compounds in the MINENV of each microorganism, indicating that the size of MINENV corresponds negatively to the breadth of environments an organism lives in, as expected (Spearman rho = −0.41, p = 1.4e-20; see Figure 3b).

We next compared the number of compounds in the MINENV of each microorganism directly against the number of ecological environments each microorganism lives in. To do this, we turned again to the Greengenes database of ecological distributions of organisms. Consistent with our observations from the lifestyle categorizations, we found that MINENV size negatively correlates (though to a weaker extent) with the number of environments that a species is found in (Spearman rho = −0.17, p = 6e-18; see Figure 3c). This upholds the general observation that environmental versatility scales negatively with nutritional fastidiousness.

Most microorganisms required 11 to 14 metabolites in their MINENVs (see Figure 3d), but there is a long tail of microorganisms that require many more metabolites. As a rule, we found that microorganisms requiring a very large number of metabolites live in a small number of ecological environments (p = 4.6e-6 in ranksum test of the number of environments for microorganisms with > =  or <20 metabolites in their MINENV, Figure 3d). Of the 33 microorganisms requiring over 35 metabolites, 14 are species of the genus *Borrelia*, which are known to have highly specific and fastidious nutritional requirements [29]; 4 have the taxonomic designation *Candidatus*, since they have not been grown in pure culture; and the rest, which include 9 species of *Mycoplasma*, are similarly either obligate intracellular bacteria or are known to have highly fastidious nutritional requirements. The microorganism with the very highest number of compounds in its MINENV is *Mycoplasma genitalium*, which is an obligate parasite with one of the most minimized genomes known. These results strongly suggest that more fastidious microorganisms tend to be more ecologically limited, and that obligate intracellular parasites have highly fastidious nutritional requirements, reflected in their MINENV size.

### Nutritional fastidiousness scales negatively with genome size and interspecies cooperation

**Figure 4:**
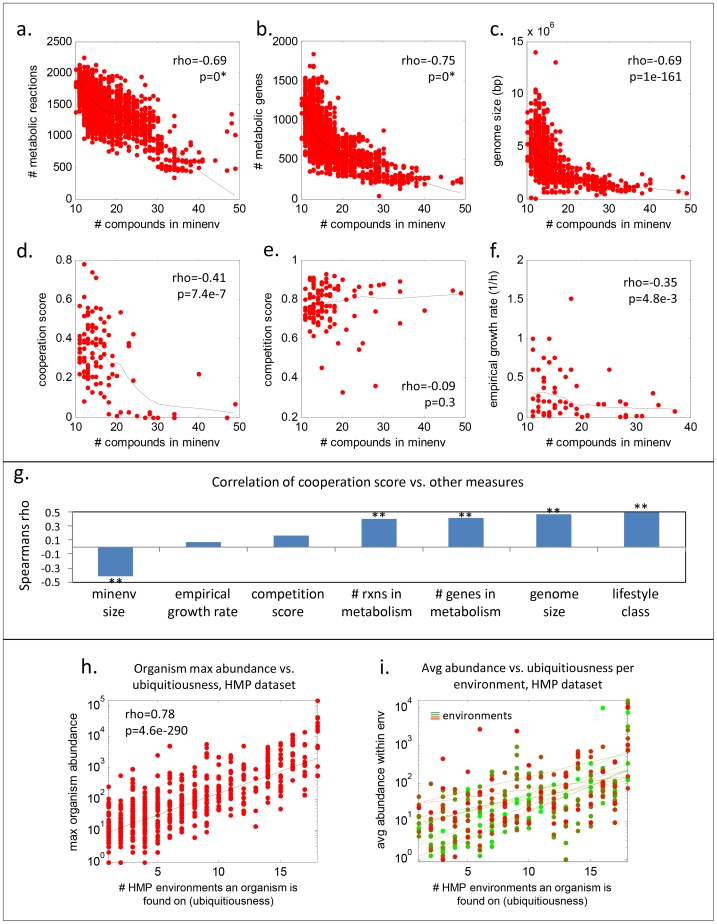
Nutritional fastidiousness scales with various ecological and lifestyle factors. The plots compare minenv size to (A) the number of metabolic reactions, (B) the number of metabolic genes, (C) the size of the genome, (D) cooperation score, (E) competition score, and (F) growth rate for all organisms we were able to map. Cooperation and competition scores were computed as averages per organism of all organism-organism scores from [7], and empirical growth rates were taken from [38]. A higher cooperation(competition) score denotes more cooperation(competition). Lines in each plot denote robust Lowess fits. (G) shows the Spearman rhos for the cooperation score against various other measures; ** denotes significance with p<1e-4. Notably, cooperation score correlates better with lifestyle class than the other metrics tested. (H) shows organism max abundance of each organism (y-axis) versus the count of environments in which the organism was present (i.e., ubiquitiousness; x-axis) in the Human Microbiome Project (HMP) dataset (from the OTU abundance table in the 2010 HMP data freeze). Dots represent individual OTUs. (I) depicts average abundances of all organisms with a given ubiquitiousness score in a given environment (dots), with a different color and a trendline for each environment. All lines in (H) and (I) are exponential fits.

Our observation that MINENV size correlates with the breadth of ecological niches led us to seek other potentially interesting variables that might be associated with nutritional fastidiousness. To do this, we compared MINENV size to several common genomic and metabolic metrics. We found that MINENV size correlates strongly negatively with several key genomic metrics, including number of metabolic reactions, number of metabolic genes, and genome size (rhos< = −0.69 for all three in Spearman test; see Figure 4a–c). Fastidiousness hence is associated with small metabolic networks and small genomes. This is plausible, considering that a genome size of at least ∼1.75 Mb is required to produce all essential compounds in a cell endogenously [30].

In addition to these genomic measures, we also checked two lifestyle measures published previously by our group: average competitiveness score, which measures the tendency of organisms to compete for resources, and average cooperation score, which measures the tendency of organisms to benefit from co-growth due to nutrient sharing (see: [7], and methods). We found that MINENV size does not correlate with organism competitiveness, whereas it does correlate negatively with cooperation (rho = −0.41, p = 7.4e-7 in Spearman test; see Figure 4d–e). The latter observation suggests that with an increased range of niches available to metabolically-versatile non-fastidious organisms, there is increased chance for mixing between different species, and hence a greater advantage in the ability to share nutrients and be more cooperative. Indeed, the cooperation score correlates more strongly with lifestyle class than with any of the other metrics we tested (see Figure 4g), which supports this hypothesis.

### Less fastidious organisms grow faster and are more abundant even within specific niches

In addition to the above correlations, we found that MINENV size correlates significantly negatively with growth rate (rho = −0.35, p = 4.8e-3 in Spearman test; Figure 4f). Thus not only are less fastidious organisms more able to share nutrients, but they also grow faster than the more fastidious ones. This suggests a mechanism that might contribute to the current distribution of organisms in niches, in which certain highly metabolically versatile organisms tend to dominate across large numbers of niches. Indeed, an analysis of organism abundances for 1408 organisms across 18 human body sites shows a strong association between the number of environments an organism is found in and the abundance of the organism on those environments (rho = 0.78, p = 4.6e-290 for max abundance vs ubiquitousness across organisms; see Figure 4i and 4j; data taken from HMP 16S data freeze in 2010; [31]). The higher growth rates of less fastidious organisms might thus contribute to their dominance even in many environments in which fastidious organisms have found their specific niches.

This observation also has broad implications for efforts to culture difficult-to-culture organisms. Aside from the fact that many of these organisms are oligotrophs and thus will require minimal media to grow at all, overly rich media will be more likely to also enable growth of more ubiquitous organisms, which with their higher growth rates will outcompete the more fastidious ones in culture. This phenomenon has been seen quite commonly in microbiology labs (e.g., fast-growing *Methanosarcina spp* will outcompete *Methanosaeta* in culture [23]), and is among other things a common cause of contamination. Developing the most minimal possible medium for a given organism is therefore a worthy goal in any effort to culture difficult-to-culture organisms. The predicted MINENVs may serve as a rational starting point for developing such media.

## Discussion

Using a simple new predictor of minimal media for microorganisms, this study explores potential new associations describing the relation between microbial nutritional needs and other central metabolic attributes on a large scale. To this end we developed MENTO, which estimates potential minimal media for thousands of microorganisms based on analysis of genome-scale metabolic models built automatically by SEED. We tested MINENVs predicted by MENTO using laboratory experiments with new designed media and by comparing it to known lab media, and also showed that the predicted nutritional needs of microorganisms that tend to co-grow in nature are more similar than for those that rarely co-grow. We then used predicted MINENVs to explore how nutritional fastidiousness relates to breadth of ecological niches, genome size, and interspecies cooperation, and identify several interesting associations that characterize their relationship. Next, we showed that fastidious organisms tend to live only in specific niches, and that they grow less abundantly than less fastidious organisms even within those same niches, This may contribute to forming the distributions of organisms among populations that are currently observed.

As nutritional yardsticks, MINENVs reveal a number of interesting ecological trends. As a source for developing usable minimal growth media, however, the MENTO method still needs further refinement. Notably, because MENTO determines the lowest number of compounds that can be used to fulfill the nutritional needs of a microorganism, it will sometimes ‘pack’ multiple nutritional needs into a single complex compound, such as including 2,3-cyclic CMP as the simultaneous carbon, nitrogen, and phosphorous source for *E. coli* (see Figure S4 in File S3). This may yield unrealistically ‘simplified’ media, which are in fact harder for an organism to grow on than if these nutritional needs were filled by multiple compounds.

Indeed, we also gain noise directly because of our use of SEED models. Although these are the only models available that are appropriate for ecological analysis across many species, they are draft models that are not as refined as manually built GSMMs, and inevitably add a significant amount of uncertainty to our analyses. Some of this noise could be eliminated in the future by re-doing model gap-filling so as to maximize the parsimony between low confidence parts of models for different species, and ensuring that differences are related to the high-confidence parts of the models, in the way that this has been shown manually for the species P. aeruginosa and P. putida [32]. This work is a large undertaking on its own and obviously beyond the scope of the current project. Because of these caveats, one must be careful in interpreting results gained from SEED models. Topological analyses or discovery of broad trends are more appropriate goals than dissection of the biology of specific organisms for new insights, especially in lower-confidence models (such as many used in this study, which are based on genomic information and computational gap-filling, with no tuning from phenotypic information). In light of this, we have focused in this work on broad trends that are likely insensitive to noise.

Despite these shortcomings, a large amount of genomic data can now be obtained for uncultivated microorganisms using single-cell genomics or metagenomics, and MENTO may be a useful tool in designing culture media for them. The fact that in lab tests our new media turned out to be less selective than desired should be acknowledged, and points to the limited accuracy of current automatically-built metabolic models. Addition of trace metabolites, as well as certain uncommon compounds (e.g., phenol and asparagines-glycine for *B. subtilis*), were required to get some of the models to display *in silico* growth on media they grew on *in vitro*. This indicates a need for careful curation of trace metabolite usage in the SEED, as well as plugging of probable gaps in some of the models. Yet MENTO-predicted media may serve as initial recommended starting points in the search for minimal media, and their overall permissiveness to growth of non-target species (as shown in Figure S4c in File S3) might be mitigated by multiple dilutions as done for Sar11 [33], or by providing additional selective constraints such as growth temperature, salinity and antibiotic supplementation, which may guarantee the survival of the desired species.

We therefore supply the MINENV predicted media as a database for use by the research community (see Table S2 in File S2). We expect that future studies will elaborate and improve on the results presented in this work, and will hopefully reveal further associations that underlie microbial distributions and growth. Some of the organisms for which we provide MINENVs are currently challenging to culture (e.g., some strains of *Prochorococcus* and *Pelagibacter*), and in addition, methods are being developed for determining full or near-full genomes of unculturable organisms [34]. With sequenced genomes of such organisms, the SEED pipeline can produce metabolic models that we can then analyze using MENTO, and hopefully speed the development of new culture media. Because of the tight connections between oligotrophy, fastidiousness, and development of new highly minimal media, MENTO may thus become a key addition towards this important and timely goal.

## Materials and Methods

### Metabolic models from SEED

We obtained 3286 strain-specific genome-scale metabolic models from SEED, spanning all microorganisms with genomes in the SEED database as of late 2011. All microorganisms could obtain biomass *in silico* when grown on rich medium. This list was shortened to 2529 models by taking only those built from the biggest genome that mapped to each taxon ID, as these typically represented the most complete genomes from multiple iterative sequencing/assembly efforts, and would therefore include fewer gap-filled (and thus low-confidence) reactions.

### MENTO: a method to formulate MINENVs and critical metabolites

MENTO is an algorithm for predicting minimal media components, which proceeds in two steps:

1. Non-unique minimal environments are determined via a mixed integer linear programming (MILP) solve for the least number of distinct metabolites that can enable production of biomass in the SEED model for an organism.
2. Unique MINENVs (these are what we refer to as ‘MINENVs’ throughout the paper) are computed through a second MILP optimization, which minimizes the sum of molecular weights of compounds in the minimal environment, while keeping the same number of compounds determined in the first MILP solve. In order to improve consistency, compounds with the same molecular weights are always chosen by MENTO in the same order.

To check that the ordering of metabolites would not lead to degenerate solutions (i.e., choosing one compound of a given molecular weight when another could have been used instead), we took the unique MINENVs we calculated from this step and searched for any single compounds from any of them that could be switched out with another single compound of the same molecular weight. Reassuringly, we found that across all of the organisms for which we calculated MINENVs, no compounds could be thus exchanged while still maintaining the minimum required biomass production.

Critical metabolites are the metabolites within MINENVs that cannot be exchanged for any other compound under any condition in which a cell can produce biomass. MINENVs and critical metabolites may be defined for different biomass cutoffs. Unless otherwise noted, a ‘nonzero biomass’ cutoff of biomass > = 0.1 was used.

The first optimization of MENTO is formulated as a mixed integer linear program (MILP) as follows:
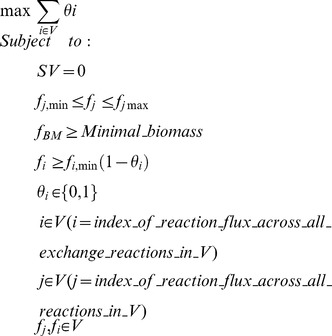
Where: ***S*** is the stoichiometric representation of the metabolic model, where the columns represent the reactions in the model. ***V*** is a vector that represents the fluxes for all reactions in the model, and ***f_j_*** represents the flux of reaction ***j*** in the stoichiometric matrix. ***f_BM_*** represents the flux of the biomass reaction in the metabolic model.

Here, in addition to the usual FBA constraints, there is:

- a constraint on minimal growth rates, ***f_BM_≥Minimal_biomass***. The minimal biomass value can be specified as a value or a certain percentage of the maximal biomass the organism can reach in an optimal media (unless otherwise specified, the biomass cutoff was a small nominal value of ***> = 0.1*** absolute flux units).
- a constraint expressing whether or not an exchange metabolite exchanged by ***f_i_*** is consumed: ***f_i_≥f_i,min_(1-ϑ_i_)***, where ***f_i_***, is the flux running through the exchange reaction ***i***, and ***f_i_≤0*** when the metabolite associated with the flux of the exchange reaction ***f_i_*** is consumed (negative flux). Here, the binary variable ***ϑ_i_*** attains a value of ***1*** if metabolite ***i*** is not consumed (***f_i_, ≥0***) by any of the organisms, and ***0*** otherwise. It should be stated that every exchange reaction is associated with a single metabolite, which can either be taken up or secreted.

Identifying a minimal set of exchange reactions and their associated metabolites in a medium then amounts to maximizing the sum of the ***ϑi*** variables over all exchange reaction fluxes in ***V***{***f_i_***}. From this calculation we get the minimal number of metabolites needed for the microorganism to grow at least with ***f_BM_***_**-**_ **≥** ***Minimal_biomass***. This minimal number of metabolites is what we use as our measure of fastidiousness, and the set of metabolites making up the minimal environment after solving are referred to as ‘non-unique minimal environments’ (since there could be various sets of compounds that could equivalently be output by the optimization). These ‘non-unique minimal environments’ are only briefly analyzed and mentioned, in preference for the MINENVs calculated in the next step.

The second optimization in MENTO is formulated as follows:
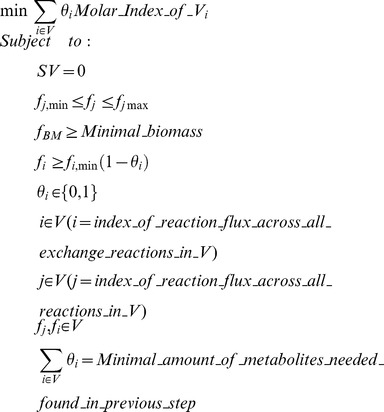
Here we have changed the objective function and added an additional constraint, which limits the number of up-taken external metabolites in the media to the number found in the previous step. The ***molar_index_of_f_i_*** is the index of the exchange metabolite in a vector where all metabolites are sorted by their molar weight in an ascending order. The output of this phase of MENTO is a list of metabolites needed for the microorganism to grow at least with ***f_BM_*** **≥** ***Minimal_biomass***. We call this list of metabolites a MINENV.

#### Computing the critical metabolites

To compute critical metabolites, we check the essentiality of each metabolite found in a MINENV, while allowing uptake from all other external metabolites. If the microorganism cannot grow in this condition, then the metabolite is essential under any condition and is thus regarded as a critical metabolite.

### Mapping SEED models to ecological environments from Greengenes

Ecological distributions of microorganisms were obtained from the Greengenes database [35] using mappings developed in [25]. Greengenes holds 16S rRNA sequences for microorganisms found in samples reported in hundreds of scientific papers. We used a sample-to-environment mapping developed in [25], in which keywords in the greengenes sample descriptions were used along with a mapping to the ENVO database [25] to determine an environment corresponding to each sample. The result of these previously developed mappings is a set of rRNA sequences, representing operational taxonomic units (OTUs), distributed among a set of ecological environments in which they were found to occur.

Sequences of 16S rRNA for all sample OTUs were mapped to microorganisms from SEED using BLASTP (with cutoffs of e-value< = 10e-10 and amino acid identity > = 99%). Finally, SEED microorganisms mapped to environmental 16S sequences were assigned to the appropriate environments. To investigate artifacts caused by mappings of multiple SEED models to a single 16S sequence, we employed certain filters in mapping species for some of our analyses. Typically, a single microorganisms was chosen to represent each 16S sequence, in order to eliminate redundancy.

### Growth on selective media *in vitro*

Media were designed based on minenv predictions, with some compound substitutions to make the media easier to produce or to make them more selective, as shown in Figure S4 in File S3. Compositions of the 5 media are shown in Table S5 in File S2. Tests of manual models were done using iMO1056 [27], iBsu1103 [36], and iAF1260 [37] for *P. aeruginosa*, *B. subtilis*, and *E. coli*, respectively. A minimal number of extra metabolites (phosphate, nh4, mn2, zn2, cu2, ca2, cl, k, mg2, cobalt2, and fe3) was added to each model in all conditions in order to allow *in silico* growth.

Bacterial species of interest (*Escherichia coli*, *Pseudomonas aeruginosa*, *Agrobacterium tumefaciens*, *Bacillus subtilis*, and *Serratia marcescens*) were grown overnight in LB to stationery phase, then washed in sterile NaCl (0.9% w/v) solution and diluted ×100 into the required media. Each species was inoculated to each of 5 specific minimal growth media as well as in LB (positive control). The bacteria/media combination was then grown overnight at 37 degrees C with shaking. Two types of growth assays were used:

- End point assays: 1 ml of each bacteria/media suspension was aliquoted to duplicate 15 ml tubes; absorbance at 595 nm was measured after 24 hours using a spectrophotometer.
- Kinetic assays. 200 ul of each bacteria/strain combination was aliquoted to duplicate wells in a 96-well plate. Plates were incubated in a Lumitron plate-reader and Abs595 absorbance recorded every 15 minutes.

### Calculating distance metrics

The typical similarity metric used in this study is the Jaccard similarity, which is calculated for two binary vectors thus:
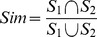
Where *S_1_* and *S_2_* represent sets of binary properties of two microorganisms. Jaccard similarities are calculated for a number of properties: notably, MINENVs (each property in the sets representing presence or absence of a given metabolite in the microorganism's MINENV) and ecological distribution (each property representing presence of a microorganism in a given environment). When other metrics are used, we describe them.

### Reconstructing known lab media from DSMZ

PDF files for the DSMZ media were downloaded from: http://www.dsmz.de/?id=441. Files were manually parsed to extract media components, and components were then manually linked to SEED compounds. The mapping of media to organisms that grow on them was kindly provided by DSMZ.

### Analysis of cooperation and competition scores for species

To obtain single scores for each species, we averaged cooperation or competition scores over all pairings with other species (118 species total) from [7].

## Supporting Information

File S1 **Supplementary text.** This contains a number of supplementary methods and results sections, as well as figure legends for the supplementary figures (in File S3).
(DOCX)

File S2 **Supplementary tables.** This contains supplementary tables S1 through S5, as referenced in the main text.
(XLSX)

File S3 **Supplementary figures.** This contains supplementary figures S1 through S8, as referenced in the main text. Legends for these figures are provided in Supplementary Text File S1.
(PPT)
