# Supplementary material for: A Novel Nutritional Predictor Links Microbial Fastidiousness with Lowered Ubiquity, Growth Rate, and Cooperativeness
Source: PLoS Comput Biol. 2014 Jul 17;10(7):e1003726. doi: 10.1371/journal.pcbi.1003726 (PMC4102436; doi:10.1371/journal.pcbi.1003726)
Supplement: File S1 — Supplementary text. This contains a number of supplementary methods and results sections, as well as figure legends for the supplementary figures (in File S3). (DOCX) [file pcbi.1003726.s001.docx]

# Supplementary Materials

Contents

[Supplementary Materials 1](#_Toc389609194)

[Supplementary Methods 1](#_Toc389609195)

[Standard bounds of SEED models and formulation of MENTO 1](#_Toc389609196)

[Calculating alternatives for non-critical metabolites 2](#_Toc389609197)

[Detailed methods for Figure 2 3](#_Toc389609198)

[Supplementary Results 3](#_Toc389609199)

[Characteristics of non-unique minimal environments, MINENVs, and critical metabolite sets 3](#_Toc389609200)

[Experimental testing of predicted MINENVs 4](#_Toc389609201)

[Organisms with similar MINENVs vs similar known lab media 5](#_Toc389609202)

[Amino acids are the most differentiating metabolites between environment aggregate media 6](#_Toc389609203)

[Extended discussion: Noise in the ecological data 7](#_Toc389609204)

[Supplementary Figure Legends 8](#_Toc389609205)

# Supplementary Methods

## Standard bounds of SEED models and formulation of MENTO

2529 metabolic models were generated by the SEED-Models metabolic models automatic generator (<http://seed-viewer.theseed.org/seedviewer.cgi?page=ModelViewer>) ([*11*](#_ENREF_11)). Briefly, in these models, a stoichiometric matrix (**S**) is used to encode the information about the topology and mass balance in a metabolic network, including the complete set of enzymatic and transport reactions in the system and its biomass reaction. Reactions are inferred from genome annotations and specialized prediction tools. Given a metabolic model, Constrained-Based Modeling (CBM) provides a solution space in terms of predicted fluxes that is consistent with the constraints set up by the model. Flux balance analysis (FBA) ([*12*](#_ENREF_12)) is a CBM method that further constrains the solution space by solving a linear problem of maximizing or minimizing a biomass production rate objective function ([*13*](#_ENREF_13)*,* [*14*](#_ENREF_14)). biomass production rate describes the rate of production of a set of metabolites required for cellular growth, where a higher biomass flux corresponds with a faster growth rate of the organism ([*15*](#_ENREF_15)). For each species we calculate its maximal biomass production rate by assuming that all exchange reactions can be potentially fully active (which is equivalent to assuming a rich media). The upper and lower bounds of exchange and non exchange reactions are conventionally set as follows:

For irreversible reactions:

Exchange reactions:

0 ≤ V_i,ex_ ≤ V_i, Max_ex_ (V_i, Max_ex_ = 1000)

Non exchange reactions:

0 ≤ V_i_ ≤ V_i, Max_ ( V_i, Max_ = 1000)

For reversible reactions:

Exchange reactions:

V_i, Min_ex_ ≤ V_i,ex_ ≤ V_i, Max_ex_ (V_i, Min_ex_ = -50 V_i, Max_ex_ = 1000)

Non exchange reactions:

V_i, Min_ ≤ V_i_ ≤ V_i, Max_ {V_i, Min_ = -1000 V_i, Max_ = 1000}

Simulations were run using the "ILOG CPLEX" linear problems solver using the "Condor" platform ([*16*](#_ENREF_16)).

## Calculating alternatives for non-critical metabolites

For each of the metabolites found to be non-essential, we check if there is a single metabolite that replaces it. This is calculated by adding a new constraint to the following MILP formulation:

The constraint forces that no uptake will be from the selected non-critical metabolite.

## Detailed methods for Figure 2

**Similarities of aggregate media (Figure 2b)**

We formed ‘aggregate MINENVs’ as the union of compounds in the MINENVs of organisms in a defined group. To test the similarity of aggregate minenvs, we formed aggregate MINENVs from two random halves of each environment, 10 times (unless otherwise noted). We then checked the distance (as number of compounds different, not Jaccard) between aggregates. We also checked distances between aggregates of random assortments of the same number of microorganisms, but chosen from outside the environment. Distances of microorganisms from within versus outside of an environment were used to determine statistical significance.

**Insideout analysis (Figure 2c-d)**

We defined aggregate MINENVs from half of the organisms in each environment, as just described. We then determined *in silico* growth on the aggregate MINENV of organisms from within and from outside the environment (but never organisms used to produce the aggregate in question). Growth was determined using FBA. The whole test was repeated 100 times (or other, as noted) for each environment, with different aggregates in each iteration from randomly chosen organisms.

DSMZ environments were analyzed the same way as MINENVs, except 80% of microorganisms within a niche were used to define the aggregate, and growth was determined by all of the media components for an organism’s DSMZ medium being present in the aggregate as opposed to by FBA. Also, aggregates were taken using the unions of DSMZ media components, not MINENVs (this is the result shown in Figure 2d).

# Supplementary Results

## Characteristics of non-unique minimal environments, MINENVs, and critical metabolite sets

As previously mentioned, minimal environments are typically not unique for a given microorganism. However, each minimal environment may be broken into two parts: a core of critical metabolites that is unique (critical metabolites, or ‘critmets’), denoting metabolites that must be present in *any* predicted growth media environment for a given microorganism (including enviroments that are not minimal), and then a periphery of replaceable metabolites, which provide essential elements to the microorganism, but are not unique (that is, other metabolites may alternatively provide these essential elements). Replaceable metabolites may include, for example, phosphate, which for many microorganisms is a potential source of the essential element phosphorous, but not the only potential source according to our analysis (which takes into account the entire SEED compound database of >16k metabolites).

Examining the range of metabolites that compose the critical and replaceable portions of MINENVs can give insight into the basic nutritional characteristics of microorganisms. For the purpose of this analysis, we call the non-unique minimal environments from the first step of MENTO as ‘Non-Unique MINENVs’ = ‘NU-MINENVs’, and analyze their properties compared to the unique versions from the final optimization in MENTO (which are, as in the rest of the paper, called MINENVs). While the replaceable members of MINENVs are unique, the replaceable members of NU-MINENVs are not. It is therefore instructive to examine the range of compounds that could be in a NU-MINENV in the replaceable portion. As an alternative to doing an exhaustive search of replaceable members of NU-MINENVs (which would be an enormous computational task), we searched for every single-metabolite substitution from the MINENV for an organism that would still produce a viable NU-MINENV, i.e., that would sustain *in silico* biomass while not increasing the number of compounds taken up. These alternative compounds, when taken with the replaceable compounds that they substitute for in the minenvs, form a unique set per species that denotes potential sources of nutrition that we predict could be, but are not necessarily, part of a minimal environment.

Each metabolite in a minimal environment provides some essential chemical element. To gain a better picture of how these elements are obtained by microorganisms, we counted the number of metabolites within critical metabolites, replaceable alternatives in NU-MINENVs, and replaceable metabolites in MINENVs that can provide them (Figure S1). The elements broke into 3 distinct groups: elements that may be provided by many different sources, elements that may be provided by varying numbers of sources in different microorganisms, and elements that are only provided one way (see Figure 2). The first category, including C, H, O, and N, counts the major constituents of cell biomass that also are present in the broadest ranges of biochemical compounds. Most commonly, microorganisms could gain each of these compounds through around 10 possible alternate replaceable metabolites, as seen in the peaks of the histograms for each of these compounds in Figure 2b (this is out of the >16k metabolites in the SEED database). The third class of elements includes the same ionic compounds always seen in critical metabolites (see Figure 1a); these are only required in trace amounts for growth of most cells. Finally, the intermediate class including S, P, and Fe, all of which are known to be important determinants of microbial distributions.

## Experimental testing of predicted MINENVs

To shed light on the categorizations of metabolites in MINENVs, we examined the critical metabolites, MINENVs, and potential replaceable metabolites in MINENVs for *Escherichia coli K12*. Figure S4a shows which metabolites fall into these categories, along with what essential nutrients are provided by replaceable metabolites. Iron can be provided in two forms (Fe2^+^ or Fe3^+^), while carbon (C), nitrogen (N), and phosphorous (P) can all be provided by any of three compounds: 2,3-cyclic CMP, 2,3-cyclic AMP, or 2,3-cyclic GMP. The inclusion of these cyclic nucleotides as opposed to, e.g., sugars, as carbon sources, is the result of a packing of multiple nutritional needs (C, N, and P) into a single compound in order to reduce the number of components. Biolog analysis confirmed that all three of these cyclic nucleotides are viable sole sources of C, N, and P for *E. coli* (Biologs provided by Microme).

To validate this MINENV formulation and compare predicted MINENVs (composed of SEED metabolites) to actual lab media (composed of common bench chemicals), we designed a lab medium for growth of *E. coli* (medium M3), using the MINENV predictions as a basis (see Figure S4b). To produce M3 with available lab chemicals, we replaced the MINENV component 2,3-cyclic AMP with adenosine. Studying other known media (e.g., M9) made it clear that certain elements required in the MINENVs would be provided as trace chemicals in the water used to mix up the media, so they did not need to be provided separately. The components in this category are: manganese, zinc, copper, cadmium, chloride, iron, and cobalt, all of which (except cobalt) we found to be critical metabolites in every single microorganism tested (See Figure S4b). The medium resulting from this work (medium M3) enables *in vitro* growth of *E. coli*.

To further assess the validity of our MINENV algorithm, we performed a similar process to that described for *E. coli* in order to develop new defined media for 4 additional lab microorganisms: *Agrobacterium tumefaciens*, *Bacillus subtilis*, *Pseudomonas aeruginosa*, and *Serratia marcescens*. As discussed in the Introduction, many of the problems concerning the in vitro growth of `unculturable' organisms arise since they are oligotrophs. Bearing this in mind, we capitalized on the leeway in the components we could choose to include in each minimal environment, to chose formulations that we predicted to be *selective* for the growth of a specific target organism to be cultured, i.e., that will only enable the chosen microorganism to grow, but not any of the others. We then grew each of these microorganisms in microwell batch cultures in each of the media to assess predictiveness (Figure S4c).

Reassuringly, each microorganism grew on the medium based on its own MINENV prediction. However, we far under-predicted growth of microorganisms on media not designed for them (only 6 of our 17 negative predictions turned out to be correct). This result may partially be a reflection of an intrinsic bias towards nutritional flexibility among microorganisms that are commonly grown in labs (such as these 5), but it also may point to trend of higher nutritional flexibility than our models predict.

## Organisms with similar MINENVs vs similar known lab media

We were interested to know if organisms with similar MINENVs tend to have more similar DSMZ media. To test this, we considered the set of 71 fully-defined lab media from the DSMZ database that corresponded to media for growth of specific strains in our SEED model collection. We compared difference metrics between all pairs of MINENVs versus all pairs of DSMZ media for the 65 unique microorganisms in this joint set (using the most minimal medium available for organisms with multiple DSMZ media listed), and found a significant albeit very small similarity between organism-organism pair-wise distances in these two datasets (Spearman rho = 0.08, p=1.5e-4). This trend somewhat increased if the MINENVs were recalculated requiring a higher biomass production in the *in silico* growth simulations (See Figure S2a for trend with a biomass cutoff of 100% and a cutoff to remove low-flux compounds from MINENVs; Spearman rho=0.18, p=9e-15, and p=4e-12 in ranksum test, grouping by low (<0.5) or high DSMZ media dist. The results for other cutoffs are shown in more detail in Figure S2b). Forcing MINENVs to have a higher biomass production increases the number of metabolites included in each MINENV (e.g., see Figure S3), which is consistent with the observation that the lab media are generally rich and not minimal.

There was of course no guarantee that the MINENVs we predict, which are not unique among minimal media, would look like the lab media, which are chemically defined but are not necessarily minimal. Nevertheless, we expected that there would be some trend of similarity of MINENVs and DSMZ media per microorganism.

To directly test this, we recalculated MINENVs for each microorganism, this time preferentially choosing compounds that are indeed present in the DSMZ media collection as nutritional sources for that strain (see methods). In order to account for concentrations of DSMZ media metabolites but not to under-value metabolites taken up in small quantities, we applied a gamma correction of 0.25 to the metabolite concentrations in DSMZ media before calculating Euclidean distance with MINENVs. The gamma correction consists of taking each concentration value in both the MINENVs and in the dsmz media to the given power (0.25, in this case), before determining euclidean distance. Comparing the re-calculated MINENV for each microorganism versus all 71 DSMZ lab media we find that MINENVs were closer to the proper DSMZ medium per microorganism than expected by chance (p=0.003 in a non parametric test of # significant p-values across the set of 71; see methods).

## Amino acids are the most differentiating metabolites between environment aggregate media

Aggregate MINENVs we predict have the potential to aid in developing environment-simulating or organism-specific media, with the ultimate goal of culturing difficult- or impossible-to-culture organisms. In order for these predicted media to be useful for this purpose, however, we need assurance that the compositions of the aggregate media indeed reflect each environment.

The Greengenes/envo environments we studied are arranged in a hierarchical (ENVO) tree, with some environments more similar to others. To investigate whether our predicted media create distinct and meaningful imprints on the environment level, we tested the dissimilarity of aggregate MINENVs between each environment-environment pairing versus dissimilarity of environments according to their arrangement in the ENVO hierarchical tree. This was done in three steps. First, we calculated pairwise distances of the 73 predicted aggregate MINENV media (hierarchical clustering of environments based on these distances is shown in Figure S6). Next, we calculated similar pairwise distances, this time using environment distances based on the hierarchical tree of environments in the ENVO database. Finally, we calculated the similarity of the results of the two tests. To eliminate bias due to environments having different numbers of organisms, we weighted compounds in aggregate MINENVs by the percent of organisms within the environment that have the compound in their MINENV, and compounds present below 20% were set to zero (See Figure S7a-b for a sensitivity analysis of this cutoff). This test achieved a small but significant Spearman correlation between aggregate MINENV distances and ENVO distances across all pairs of environments (rho=0.18, p=2.4e-21; see Figures S7c, see also Figure S7c), confirming that aggregate MINENVs recapitulate some part of the known similarities between environments, although with a signal admittedly not as strong as one may expect.

To better understand how the aggregate MINENVs may be leveraged to develop new selective growth media, we examined the metabolites that differentiated most strongly between environments. We clustered the environments into two groups (shown in Figure S6), and ranked metabolites from the most differentiating between the clusters to the least informative (we also re-confirmed this ranking by comparing it to the ordering of weights on metabolites in the first principle component of the environments-compounds matrix; the orderings from the principle component and from the clustering obtained a correlation of 0.9). Strikingly, amino acids were highly enriched as the differentiating metabolites. We confirmed this observation using a ranksum test on the differentiation ranking for amino acids vs. non amino acids, and found strong enrichment of amino acids as the differentiating metabolites (p=1.8e-6 for amino acids; p=2.1e-3 for dipeptides alone; and p=9.3e-9 including amino acids and dipeptides).

Amino acids are highly abundant within living organisms, but are not as freely available in most natural environments. Therefore, we hypothesized that amino acids would be the most distinguishing metabolites between ecological classifications, and specifically that they would distinguish obligate pathogens from other lifestyle groups. To check this, we formed aggregate MINENVs for each lifestyle group as described above for Greengenes, and performed principal components analysis to reduce the compound space contributing to differentiating the groups. Finally, we examined only the top two principal components, which contained 68.5% and 17% of the differentiating information between aggregate media, respectively. We found that indeed, the first principal component is highly enriched in amino acids and dipeptides, and that it clearly separates obligate parasites from the other categories (see Figure S8a-b). In particular, isoleucine, valine, arginine, and methionine showed up in the top differentiating metabolites in both analyses (between Greengenes environments and between ecological classifications). This evidence, along with our earlier observation that amino acids are more common in MINENVs than in DSMZ media but are still quite often used as viable media components (see Figure 1a and full listing in Table S1), pits amino acids and dipeptides as strong and potentially overlooked candidate metabolites for developing selective growth media in the future.

It was important to check how specific our predictions were for particular amino acids. To do this, we attempted single-metabolite substitutions for every appearance of an amino acid in any of the MINENVs, and checked how often a different amino acid could replace the original one. We found that the most replaceable metabolites were the smallest ones (alanine, glycine, proline, and serine), but the rates of substitution were surprisingly low (only 336 viable substitutions were seen among minenvs out of 54,320 possible for all amino acid combinations; see Table S4). The higher replaceability of small amino acids may partially be due to the preference for low molecular weight compounds built into the calculation of MINENVs, but the low overall number of substitutions suggests that in general, amino acid preferences we predict among microbes are specific.

## Extended discussion: Noise in the ecological data

From the ecological perspective it is notable that aside from the finding that MINENVs show greater similarity of microorganisms within versus between environments, the signals displayed in our analyses contend with a great deal of noise. For example, Figures 3b and 3c show a significant number of environments in which the distance of aggregate media is greater within the environment than between microorganisms outside the environment, contrary to the general trend and to all expectations. There are several reasons for this apparent noise:

First, our work required us to forge a connection between environmental OTUs and specific strains of microorganisms we have in our SEED model list, which is an imperfect mapping no matter how it is done. It is well established that related strains and species often vary in their metabolic networks, primarily due to lateral gene transfer, which facilitates the acquisition of metabolic novelty ([*29*](#_ENREF_29)). We experimented with a number of mappings, and found that some of our noise comes from this and is unavoidable.

Second, as noted in the main discussion, we gain noise directly because of our use of SEED models. Some of this noise could be eliminated in the future by re-doing model gap-filling so as to maximize the parsimony between low confidence parts of models for different species, and ensuring that differences are related to the high-confidence parts of the models, in the way that this has been shown manually for the species *P. aeruginosa* and *P. putida (*[*30*](#_ENREF_30)*)*.

And finally, there is the Baas-Becking hypothesis, that “Everything is everywhere, but the environment selects” ([*31*](#_ENREF_31)). Although this concept was formulated in reference to bacterial species, it may equally well apply to metabolites. Certain environments are certainly noted for particular sets of nutrients, but determining the nutrient-microorganism relationships at a metabolic modeling level is quite a challenge when it is realized that a vast array of nutrients are likely present in most environments on earth in which microorganisms thrive, and that simple concentration cutoffs will not work since essential nutrients can range in needed concentrations by over six orders of magnitude. Future studies should focus on trying to understand what compounds are present at high concentrations versus low ones, and also might benefit from focusing on very specific environments with known metabolic profiles.

# Supplementary Figure Legends

**Figure S1. Ranges of nutritional sources of chemical elements.** The number of metabolites that can provide different nutritionally essential chemical elements is listed for (a) critical metabolite sets, (b) non-critical portions of non-unique minenvs (i.e., before the 2^nd^ optimization), and (c) non-critical metabolites in minenvs. Each row represents a histogram of the percentage of organisms that can provide the given element with varying numbers of metabolites (the y-axis in each plot represents the # of providing metabolites). Elements are classed into 3 categories: ‘copious’ (i.e., these elements can be provided by a rich assortment of metabolites); ‘mid’ (i.e., provided by a few metabolites); and ‘only supplied 1 way’ (i.e., only supplied by one known metabolite).

**Figure S2. Correlation of minenv similarity and DSMZ media similarity.** For all available organism-organism pairs, we computed pairwise similarity of DSMZ media and also of minenvs, using jaccard metrics. We then checked the correlation of these two measures after re-calculating minenvs using a variety of biomass requirement cutoffs (e.g., computing a minenv when requiring the usual nominal biomass requirement per organism, or increasing the requirement to various percentages of the max achievable biomass given a rich environment, per organism). (A) displays the distances for all org-org pairs given a stringent biomass cutoff of 100%, and imposing a cutoff to eliminate accounting for low-flux compounds (this is referred to as an ‘epsilon’). (B) displays how the correlation coefficient (Spearman) is affected by biomass cutoffs and various epsilons.

**Figure S3. Effect of biomass cutoff on minenv size.** Minenvs may be re-computed using higher biomass requirements than the typical nominal one (which is 0.1 absolute flux given standard modeling conditions). Here we explore how the number of (A) critical metabolites and options for swaps of non-critical metabolites in minenvs, and (B) the number of metabolites in minenvs scale with different biomass requirements.

**Figure S4. Culturing common bacteria in minenv-inspired media.** (A) The minenv for *E. coli* is explored, along with (B) its in vitro implementation (i.e., its conversion to a producible lab medium). (C) displays the growth of 5 organisms *in vitro*, *in silico* (using SEED models), and *in silico* (using manually built GSMs available for 3 of the organisms).

**.Figure S5. Aggregate minenvs distances, groups of 5 organisms.** Distances between pairs of aggregated minenvs composed of minenvs from 5 organisms within (x-axis) or from outside of (y-axis) an environment are shown. Distances are calculated as the number of metabolites differing between a pair of aggregates. For each environment, different sets of 5 organisms are chosen randomly, and the mean and errorbars on represent variance for different sets, with each point denoting an environment. Most of the dots are above the 50/50 line (green), denoting that distances are higher for aggregate minenvs from outside-of-environment organisms than within-environment organisms. This analysis is analogous to that done in Figure 2b, except aggregates are built from groups of 5 organisms rather than from half the # of organisms listed in each environment.

**Figure S6. Clustering of environments based on aggregate minenvs.** Minenvs were aggregated for each ecological environment, and environments were subsequently clustered. The tree of environment similarity, based on this clustering, is shown in the figure.

**Figure S7. Distances of environments based on minenvs vs. ENVO hierarchy.** The plot in (C) shows a 2-d histogram of pairwise distances between ENVO environments, with distances calculated from the hierarchical ENVO environment relationship tree (x-axis of subfigure C) and the tree of aggregate minenv similarities (y-axis of subfigure C). Distances were determined after applying a cutoff to the minenv aggregates, in which compounds present in the minenvs of fewer than a defined percent of organisms within the environment (i.e., defined by the cutoff) were set to zero in the aggregate for the purpose of the analysis. (A) and (B) show the sensitivity of the rho and pval in Spearman test of the ENVO vs. minenv distances to changes in the cutoff. The correlation of 0.18 displayed in (C) is observable by eye on the 2-d histogram.

**Figure S8. Differentiating metabolites for lifestyle-based minenv aggregates.** Minenvs were aggregated for all organisms in each of 5 lifestyle groups, as described in the main text. Principal component analysis was then done between the aggregate minenvs of the lifestyle groups. (A) the 5 lifestyle groups are plotted on a principal component 1 (PC1) vs. PC2 plot. (B) Compounds with the most dominant contributions to PC1 and PC2 are listed. These compound lists are heavily enriched for amino acids and dipeptides.
