# Supplementary material for: A Novel Nutritional Predictor Links Microbial Fastidiousness with Lowered Ubiquity, Growth Rate, and Cooperativeness
Source: PLoS Comput Biol. 2014 Jul 17;10(7):e1003726. doi: 10.1371/journal.pcbi.1003726 (PMC4102436; doi:10.1371/journal.pcbi.1003726)
Supplement: File S3 — Supplementary figures. This contains supplementary figures S1 through S8, as referenced in the main text. Legends for these figures are provided in Supplementary Text File S1. (PPT) [file pcbi.1003726.s003.ppt]

## Slide 1
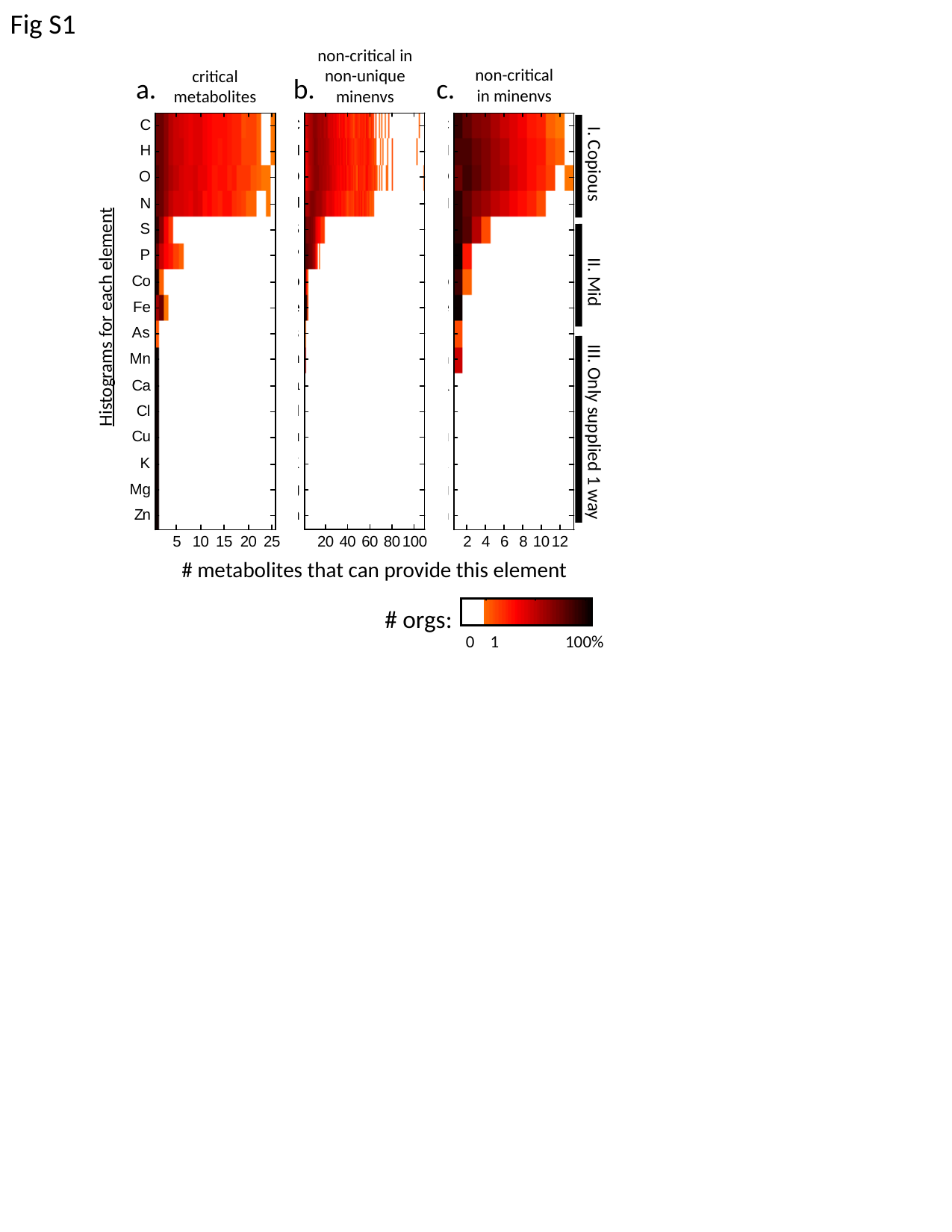

Fig S1
non-critical in non-unique minenvs
non-critical in minenvs
critical metabolites
a.
b.
c.
I. Copious
II. Mid
Histograms for each element
III. Only supplied 1 way
# metabolites that can provide this element
# orgs:
0 1 100%

## Slide 2
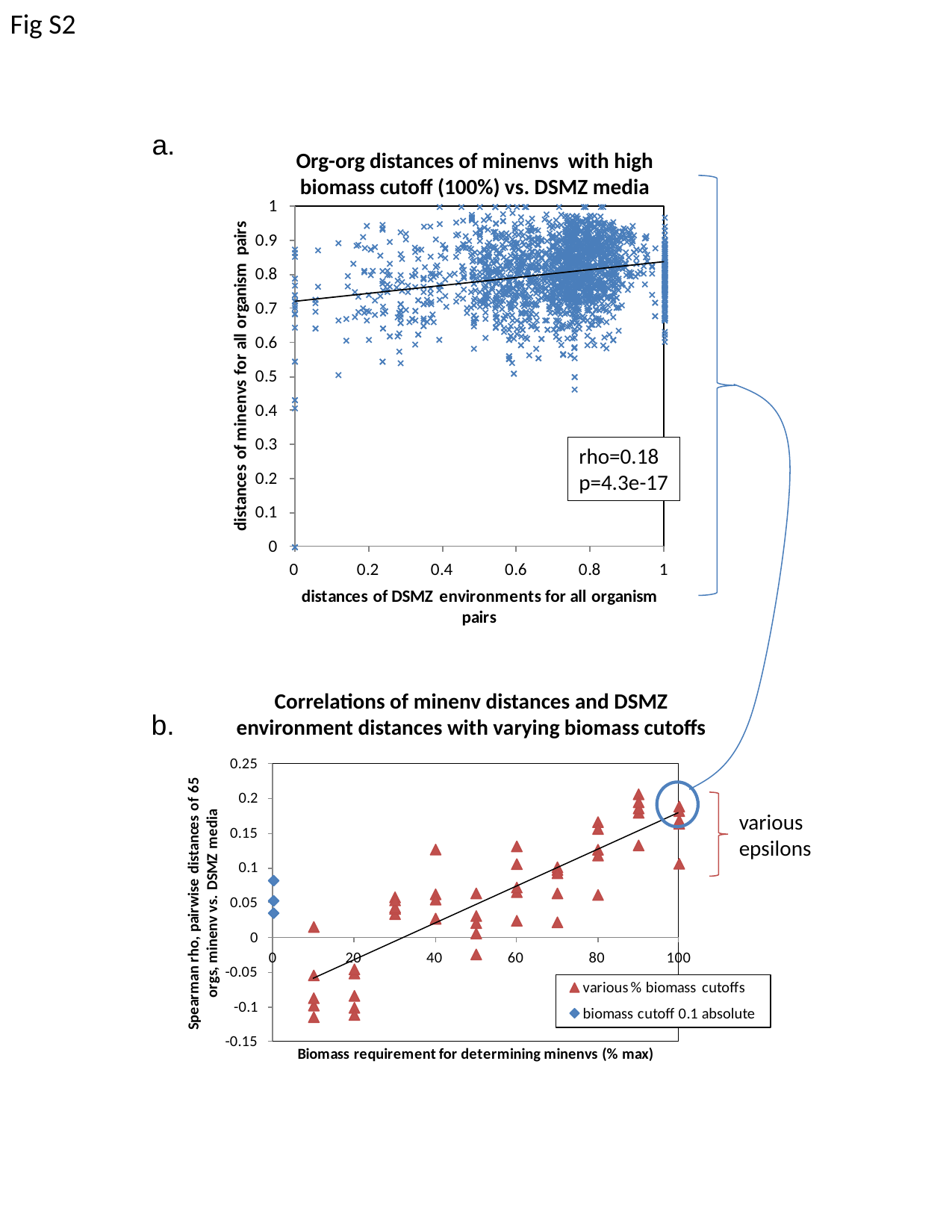

Fig S2
a.
Org-org distances of minenvs with high biomass cutoff (100%) vs. DSMZ media
rho=0.18
p=4.3e-17
Correlations of minenv distances and DSMZ environment distances with varying biomass cutoffs
b.
various epsilons

## Slide 3
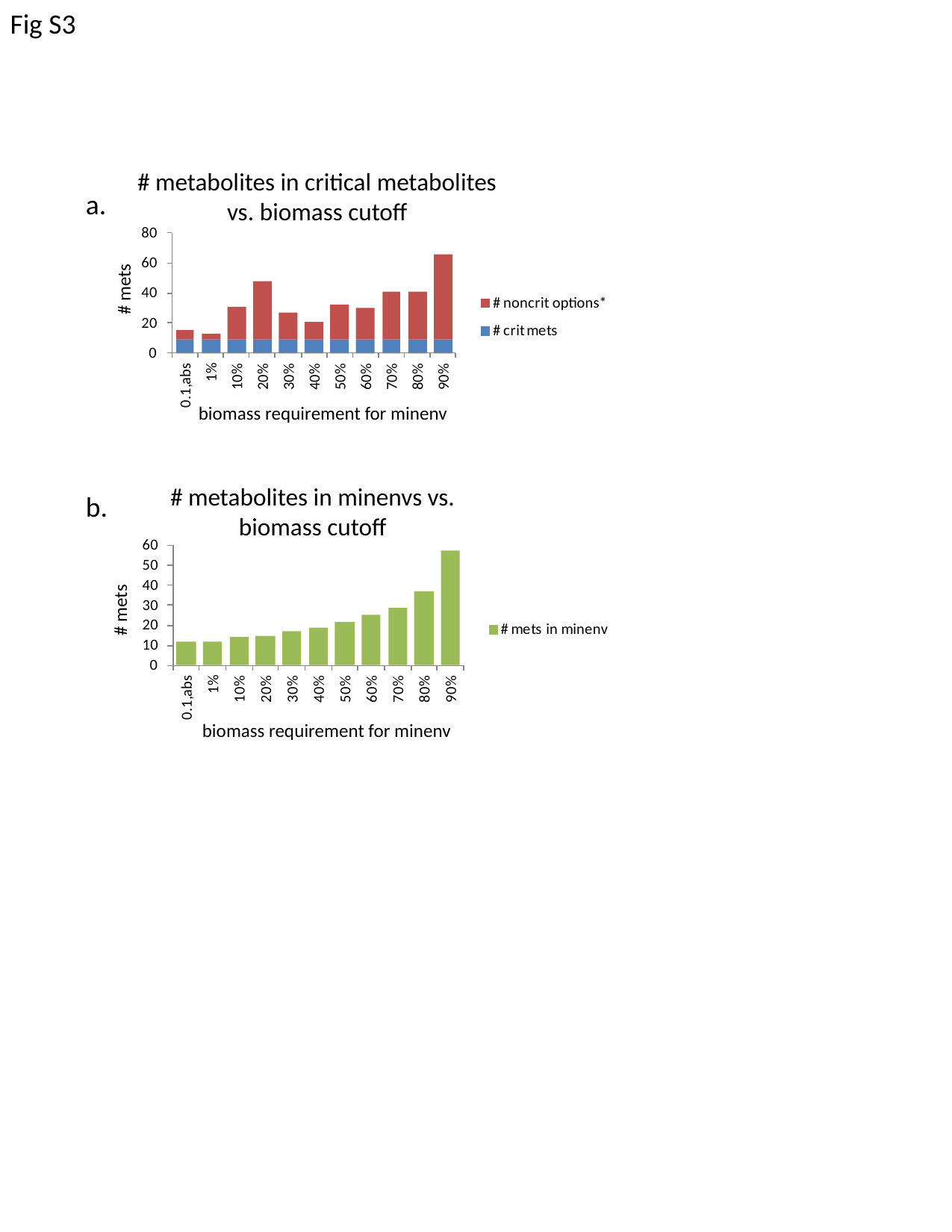

Fig S3
# metabolites in critical metabolites vs. biomass cutoff
a.
# mets
biomass requirement for minenv
# metabolites in minenvs vs. biomass cutoff
b.
# mets
biomass requirement for minenv

## Slide 4
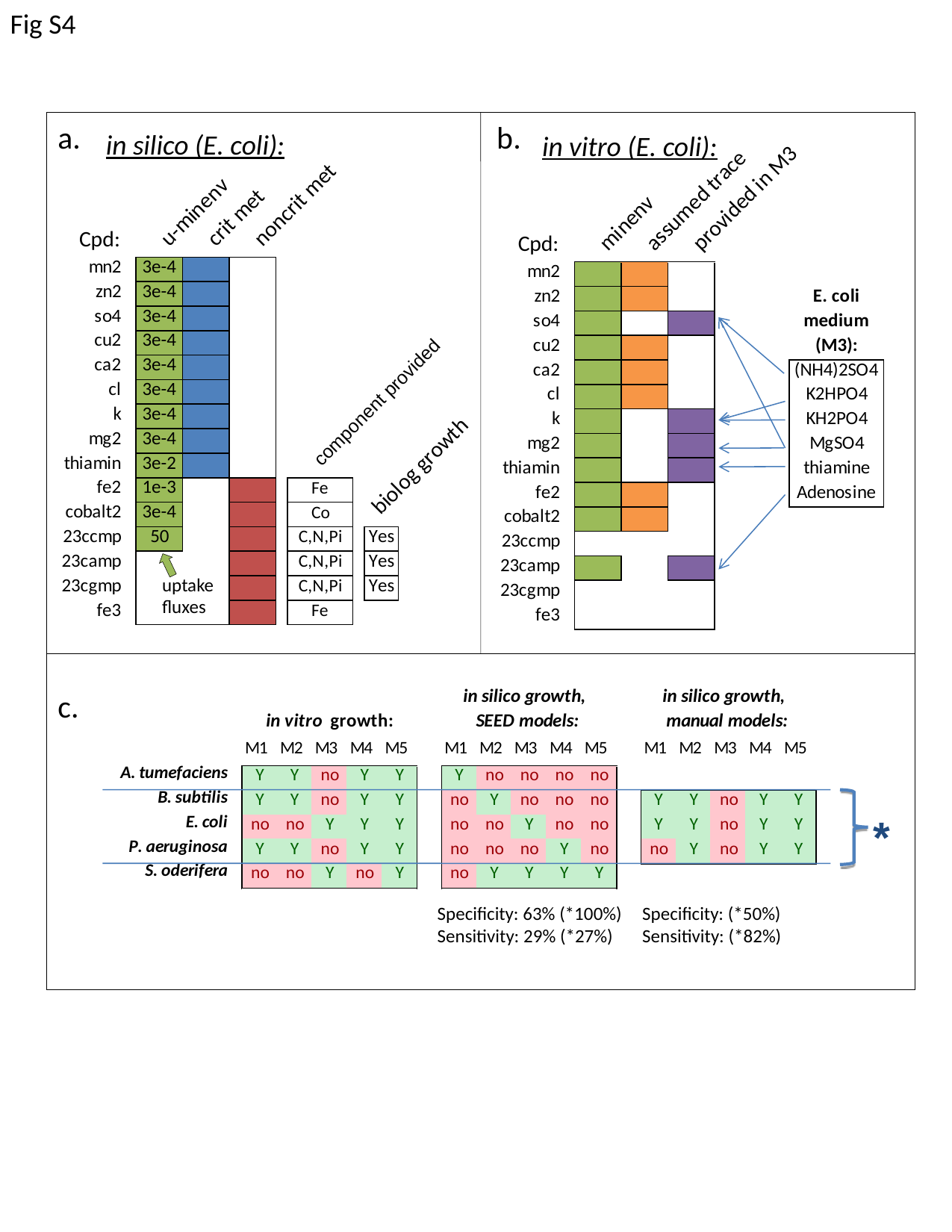

Fig S4
a.
b.
in silico (E. coli):
in vitro (E. coli):
component provided
uptake
fluxes
c.
*
Specificity: 63% (*100%)
Sensitivity: 29% (*27%)
Specificity: (*50%)
Sensitivity: (*82%)

## Slide 5
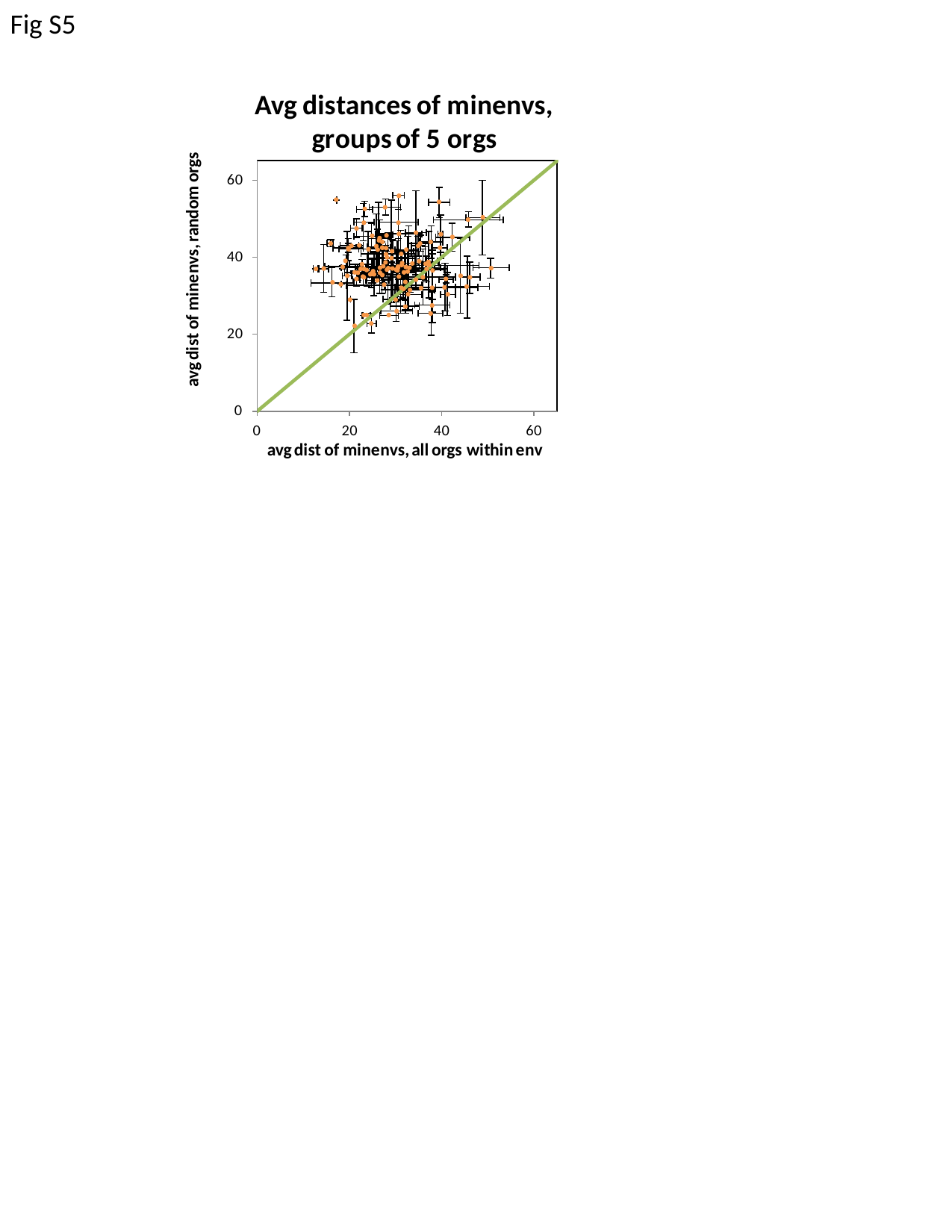

Fig S5

## Slide 6
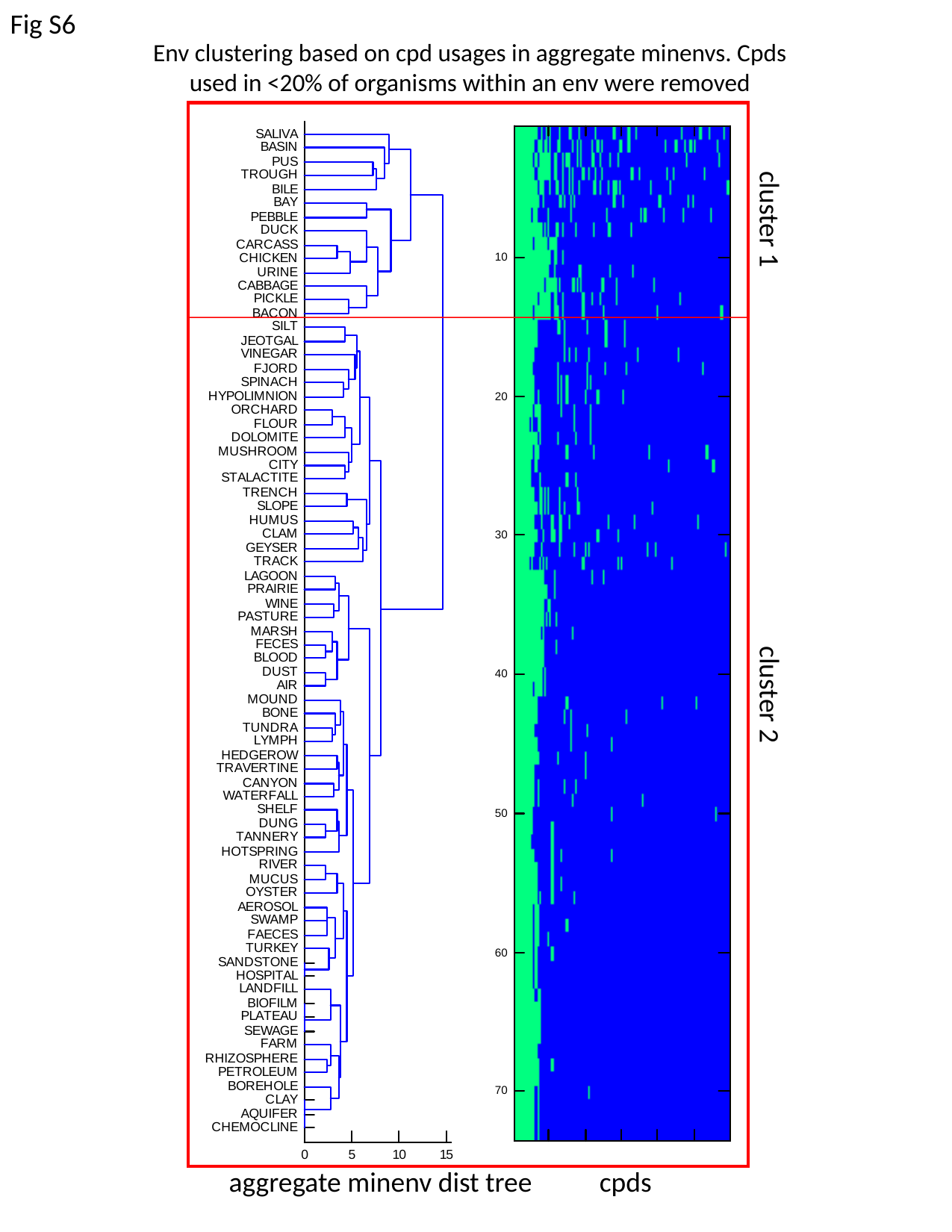

Fig S6
Env clustering based on cpd usages in aggregate minenvs. Cpds used in <20% of organisms within an env were removed
cluster 1
cluster 2
aggregate minenv dist tree
cpds

## Slide 7
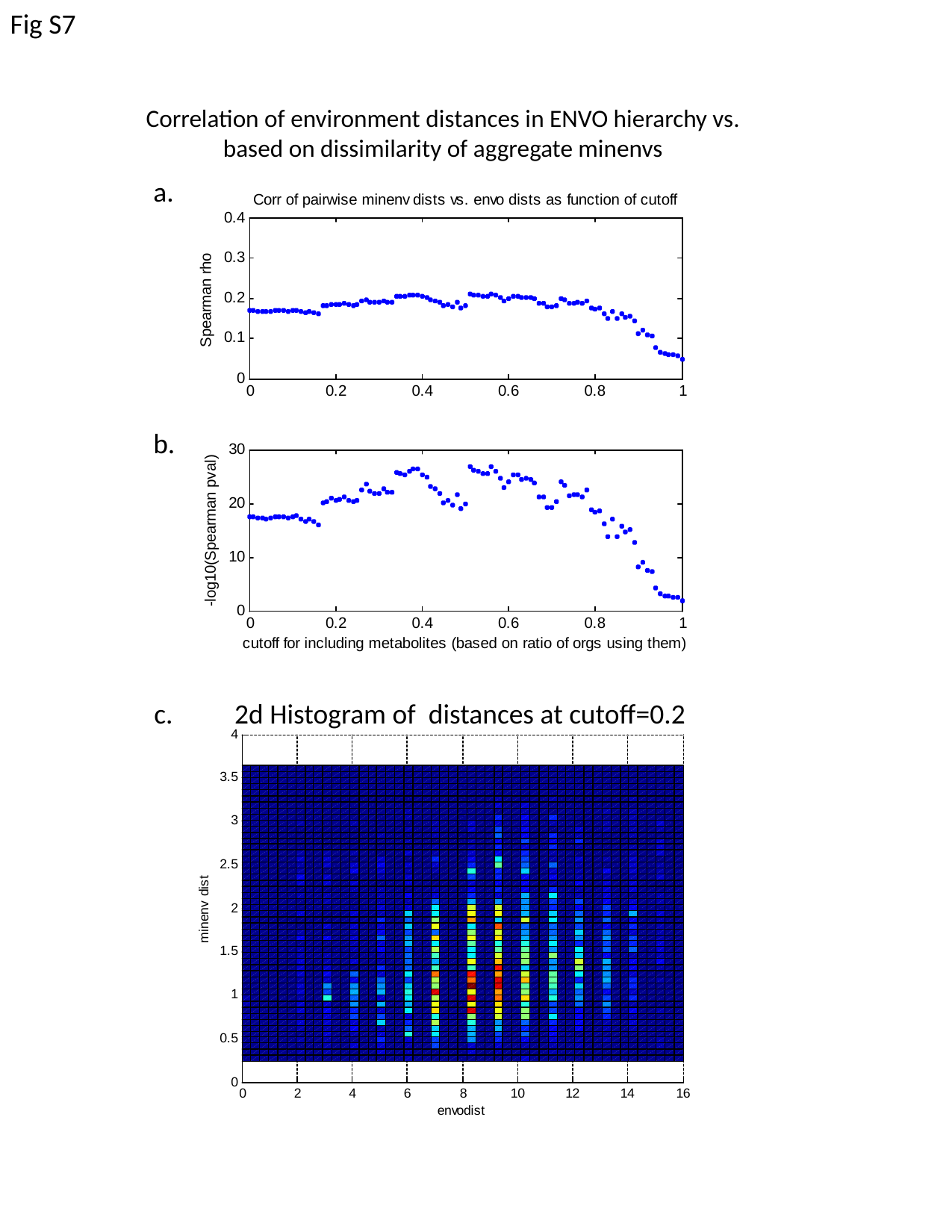

Fig S7
Correlation of environment distances in ENVO hierarchy vs. based on dissimilarity of aggregate minenvs
a.
b.
c.
2d Histogram of distances at cutoff=0.2

## Slide 8
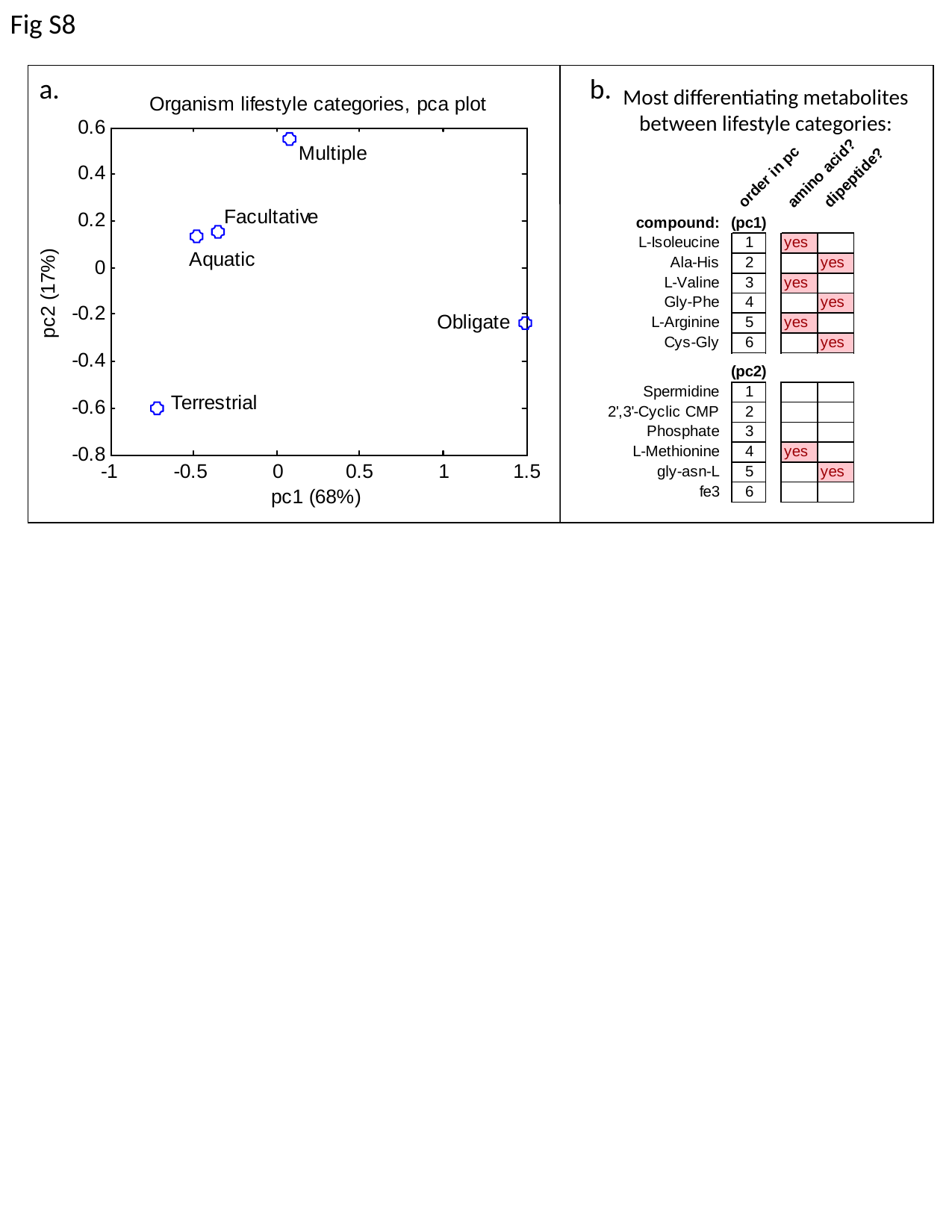

Fig S8
a.
b.
Most differentiating metabolites between lifestyle categories:
